# Bio-Inspired Explainable Evolutionary Rule Mining for Thermodynamic Performance Assessment of a Solar Greenhouse Dryer

**DOI:** 10.3390/biomimetics11070478

**Published:** 2026-07-09

**Authors:** Mehmet Das, Ebru Akpinar, Ferdi Dogan, Oguzhan Pektezel, Mithat Simsek, Sinan Akpinar, Suna Yildirim, Bilal Alatas

**Affiliations:** 1 Department of Mechanical Engineering, Faculty of Engineering, Firat University, Elazig 23119, Türkiye; 2 Department of Computer Engineering, Faculty of Engineering, Adiyaman University, Adiyaman 02040, Türkiye; 3 Department of Mechanical Engineering, Faculty of Engineering, Balikesir University, Balikesir 10145, Türkiye; 4 Department of Mechanical Engineering, Faculty of Engineering, Tokat Gaziosmanpasa University, Tokat 60250, Türkiye; mithat.simsek@gop.edu.tr; 5 Department of Physics, Faculty of Science, Firat University, Elazig 23119, Türkiye; sakpinar@firat.edu.tr; 6 Department of Software Engineering, Faculty of Engineering and Natural Science, Malatya Turgut Ozal University, Malatya 44920, Türkiye; 7 Department of Software Engineering, Faculty of Engineering, Firat University, Elazig 23119, Türkiye; balatas@firat.edu.tr

**Keywords:** bio-inspired evolutionary computing, CRb-SPEA2, explainable artificial intelligence, solar greenhouse drying

## Abstract

This study investigates the thermodynamic and drying performance of a greenhouse dryer integrated with a parabolic trough solar collector (PTSC) and develops interpretable operating rules using a bio-inspired explainable artificial intelligence framework. Outdoor apple-drying experiments were conducted, and system performance was evaluated in terms of energy, drying, and exergy efficiencies. The experimental results indicated that energy efficiency ranged from 17.7% to 29.2%, drying efficiency from 1.0% to 9.7%, and exergy efficiency from 5.6% to 8.4%. Measured variables, including temperature, relative humidity, product weight, and solar radiation, were used to classify the efficiencies into low, medium, and high categories using the Chaotic Rule-based Strength Pareto Evolutionary Algorithm 2 (CRb-SPEA2). As a bio-inspired evolutionary computing approach, CRb-SPEA2 employs population-based search, selection, Pareto dominance, and multi-objective optimization mechanisms inspired by natural evolutionary processes. In contrast to conventional black-box machine learning models, the proposed method extracts explicit decision rules that define physically meaningful operating ranges. The maximum recall values were 0.952, 1.000, and 0.971 for the high-energy-, drying-, and exergy-efficiency classes, respectively. The extracted rules identified solar radiation, temperature, relative humidity, and product weight as dominant factors affecting dryer performance.

## 1. Introduction

Energy has been a fundamental driver of industrial development and economic advancement across human history [1]. Over recent decades, rapidly growing energy requirements in industrial and commercial sectors have been largely met through fossil-fuel-based technologies, leading to increased atmospheric pollution, climate change, and adverse health impacts, thereby highlighting solar energy as a sustainable alternative for reducing fossil fuel dependence while satisfying sectoral energy demands [2]. As conventional energy reserves continue to decline, alternative energy technologies—especially solar-based systems—have become increasingly prominent in heating, desalination, refrigeration, industrial operations, and electricity production, although further advancements in high-efficiency approaches are necessary to enhance overall energy harvesting [3]. The Sun emits an enormous amount of energy, with approximately 1.7 × 10^14^ kW of solar irradiation reaching the Earth’s surface, a magnitude so large that harnessing it for only 84 min would be sufficient to meet the annual global energy demand [4].

A solar collector can be defined as a system that intercepts incident solar radiation and converts it either into thermal energy for a working fluid, as in concentrated solar power systems, or into electrical energy, as in photovoltaic applications [5]. PTSCs are lightweight and cost-effective systems capable of achieving high operating temperatures with favorable efficiency, in which a metal absorber tube enclosed by a glass cover is positioned along the focal line to minimize thermal losses [6]. The parabolic trough collector system employs a parabolic mirrored reflector to concentrate direct solar radiation onto a tubular absorber positioned along the focal axis of the parabola [7]. When incoming solar radiation reaches the parabolic trough collector, it is optically concentrated along the focal axis, captured by the receiver, and converted into heat energy that is subsequently delivered to the collector receiver tube [8]. Ongoing advancements in mirror and receiver design, alternative heat transfer fluids, thermal energy storage strategies, and process engineering, together with extensive operational experience and sustained research efforts, have established PTSC technology as the most technically developed and commercially mature option among concentrating solar power technologies [9].

Various thermal performance evaluation and machine learning application studies on PTSCs have been conducted in the literature. Benmenine et al. [10] conducted experiments to examine the thermal characteristics of a direct solar drying system by comparing a conventional configuration with a modified system incorporating a parabolic trough solar concentrator and a heat exchanger. The highest drying performance was obtained at 0.007 kg/s mass flow of the heat transfer fluid, where the drying air temperature increased to 6–11.3 °C, a greater value than the surrounding conditions compared with 4–10.0 °C for the traditional dryer, corresponding to a performance improvement of 37.6%, while a drying rate of 0.732 kg water/h·kg (dry matter) and a moisture reduction of 76.7%wb were achieved within 4.5 h. Ghafari and Behzad [11] examined the drying behavior of a solar dryer employing a parabolic trough collector that transfers thermal energy to airflow through water circulation and metallic spiral tubes. Both collector configurations shortened the drying duration by 7 h relative to conventional sun drying, while the hybrid flat-plate and parabolic trough arrangement enhanced efficiency by 8%, decreased the moisture ratio by 18%, increased the rate of drying by 6%, achieved a daily temperature rise of 11–16 °C compared to 5–14 °C for the single arrangement, resulted in the average cost of heat calculated at 0.16 USD/kWh, and reduced CO_2_ emissions by 104–219 kg per year. Das et al. [12] experimentally investigated a greenhouse-type drying system assisted by a PTSC and a dual-axis tracking mechanism. They reported that solar tracking improved the energy, exergy, and drying efficiencies and significantly reduced the drying time. Salah et al. [13] conducted experiments to test a solar drying system with a dual-axis tracking feature and a cylindrical–parabolic solar concentrator. Using the system, 10 kg of apricots underwent drying, with moisture content dropping from 69% to 8% (wb). In addition, the system reached temperatures of 115 °C at the receiver and 70 °C in the drying compartment, achieved an average thermal efficiency of 25.93% and a maximum efficiency of 45.25%, reduced the moisture content by 71.76%, and resulted in a payback period of 0.43 years. Pakouzou et al. [14] created a numerical simulation of a parabolic trough solar concentrator intended to be integrated with an indirect solar dryer and analyzed its performance using energy, exergy, and environmental indicators. For an arrangement with a 2.2 m length, a mass flow of 0.07 kg/s, and an entry temperature range of 32–36 °C, the model predicted outlet temperatures of approximately 70 °C (the biggest) and 62 °C (average of the month), average emissions of 302.02 gCO_2_ per month and 9.93 gCO_2_ per day, and annual CO_2_ emissions of 3624.27 gCO_2_, compared with 7618.25 gCO_2_/year for solar thermal systems and 19,605.78 gCO_2_/year for biomass-based drying technologies. Büker et al. [15] experimentally investigated a solar-assisted rotary desiccant-based dehumidification system coupled with a parabolic trough solar air collector for dehumidification applications. The system supplied a maximum regeneration air temperature of 45.8 °C at an air volume flow of 60 m^3^/h, achieved the highest dehumidification efficiency at a regeneration air volume flow of 80 m^3^/h, increased the process air inlet temperature by 4–6 °C, and reduced the regeneration air temperature by 10–15 °C. Das et al. [16] experimentally analyzed the energy, exergy, and drying characteristics of a greenhouse drying system integrated with parabolic corrugated vacuum tubes and established prediction models using machine learning approaches. The system exhibited energy efficiencies between 7–33.4%, exergy efficiencies of 4–7.4%, and a drying efficiency of 61.5%, while the SVM algorithm demonstrated superior predictive capability with test MAE/RMSE values of 0.0013/0.0035 for exergy efficiency, 0.0037/0.0120 for energy efficiency, and 0.0046/0.0066 for drying efficiency. Akhijahani et al. [17] investigated an indirect solar drying system integrated with a PTSC, air recirculation setup, phase change material, and Al_2_O_3_ nanofluid (3.75%) for drying 5-mm rhubarb slices. By applying inlet air recirculation ratios of 0–25–50–70%, the total drying efficiency was enhanced by 2.32–8.21%, energy consumption decreased by 1.91%, the exergetic efficiency varied between 35.4% and 61.3%, and the drying duration was significantly reduced with rising air recycling. Ullah and Kang [18] experimentally examined the dehydration behavior of a PTSC for drying seasonal fruits under different air mass flow rates and absorber pipe diameters at a concentration ratio of 60. The maximum mean efficiency reached 23% at an air flow of 3.50 kg min^−1^ with a 5.08 cm absorber diameter, while the lowest efficiency was 19.6% at 1.5 kg min^−1^, with peak solar irradiance of 752 kJ m^−2^ h^−1^, ambient temperature of 43 °C, drying chamber and absorber temperatures of 51 °C and 84 °C, relative humidity reduced to 8%, and moisture content decreased to 8% within 11 h. Li et al. [19] analyzed the performance of a PTSC equipped with six receivers incorporating porous media with stepwise porosity and applied machine learning models to predict the receiver outlet temperature. In the semi-porous configuration, augmentation of absorber tubes increased thermal entropy production by 18.4% while decreasing heat convection coefficient and entropy generation due to friction by 16.6% and 9.2%, respectively, the use of semi-metal foam enhanced coefficient of heat transfer by 13.06% in comparison with a fully porous medium, and the ANN model achieved prediction metrics of R^2^ = 0.999, MAE = 56 × 10^−4^, RMSE = 131 × 10^−4^, and MAPE = 50 × 10^−4^. Alhamayani [20] optimized the functioning of a parabolic trough collector by analyzing hybrid nanofluids with different Al_2_O_3_ and MWCNT volume fractions and developed data-driven models to estimate the exit temperature. The maximum mean thermal efficiency of 70.54% was achieved using a nanofluid composed of 2% Al_2_O_3_ and 1% MWCNT in Syltherm-800, while the ANN model yielded prediction metrics of R^2^ = 99.99%, MAPE = 4.8 × 10^−5^, RMSE = 0.012, and MAE = 0.0057. Recent studies have also shown that the integration of renewable energy technologies with intelligent and energy-efficient computational approaches can improve system-level energy management, operational stability, and decision-making performance in solar-based applications [21,22].

The primary objective of this study is to experimentally evaluate the energy, exergy, and drying performance of a PTSC-assisted greenhouse dryer and to derive physically interpretable operating rules using an explainable artificial intelligence approach. The novelty of this research lies in transforming experimental drying data into explicit decision rules that identify optimal operating ranges for achieving high energy and exergy efficiencies and high drying efficiency. Numerous machine learning algorithms have been used to predict the performance of solar drying systems, but many of these approaches rely on black-box models and fail to fully explain the physical relationships between attributes. Classical rule extraction methods often employ deterministic or greedy search strategies, making them ineffective for optimizing multiple conflicting objectives during rule extraction. The CRb-SPEA2 framework attempts to eliminate these limitations by combining multi-objective evolutionary optimization with explicit rule mining. Furthermore, the integration of the Tent chaotic map increases population diversity during evolutionary searching, reducing early convergence and improving rule space exploration. As a result, the proposed framework can simultaneously optimize multiple classification objectives while generating interpretable rules. Consequently, the proposed framework is able to optimize multiple classification objectives simultaneously while generating interpretable rules, and provides both predictive information and practical guidance for the design and operation of solar-powered greenhouse drying systems.

## 2. Materials and Methods

### 2.1. Experimental System

**Figure 1:**
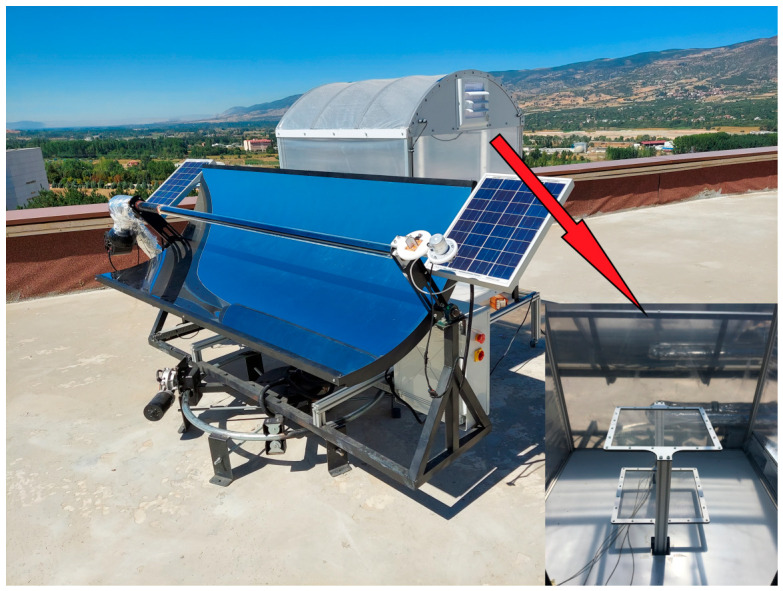
Experimental greenhouse dryer system.

Figure 1 shows the PTSC greenhouse dryer used in the experiments. In this research, the drying behavior of apple slices was examined using a greenhouse dryer integrated with a PTSC.

The experimental study was conducted in Tokat, Türkiye, which is situated between 39°51′–40°55′ N latitudes and 35°27′–37°39′ E longitudes. According to the Köppen–Geiger climate classification, the experimental site exhibits a hot-summer Mediterranean climate, which is characterized by hot, dry summers. The parabolic trough solar collector employed in this study was oriented along the North–South axis to maximize the solar radiation efficiency during the drying experiments. The samples were prepared as hollowed, oval-shaped slices with a uniform thickness of 15 mm and an initial mass of approximately 150 g, and they were evenly distributed on the drying trays. The drying trials were terminated once the change in sample mass during consecutive measurements decreased to less than 0.5 g. Determining the completion of drying based on mass stabilization over a defined time interval is a commonly adopted methodology. When the recorded mass variation remains within a narrow margin, generally ±0.5 g, the material is considered to have reached its equilibrium moisture content, indicating that further moisture removal is negligible. Applying a 0.5 g criterion ensures high measurement accuracy and repeatability, particularly in systems equipped with load-cell sensors. This approach also contributes to the standardization of experimental procedures, facilitates comparison between different tests, and prevents unnecessary over-drying of the samples. The experimental campaign was conducted between August 15 and 17, 2023, during which meteorological factors, including surrounding temperature, relative humidity, velocity of wind, and solar irradiance, exhibited comparable values. Drying experimental studies were performed over three consecutive days, and the reported outcomes represent mean values obtained from these trials. As illustrated in Figure 1, the drying system incorporates a high-efficiency solar collector based on an advanced vacuum tube configuration created to intensify solar radiation and transform it to thermal energy. A centrally positioned copper tube enhances heat transfer through direct interaction with the airflow. Air circulation is maintained by a high-capacity fan, while the heated air is conveyed through 100 mm diameter ducts into a purpose-built drying room where the apple cuts are loaded. This integrated configuration enables effective utilization of solar energy and supports a sustainable and energy-efficient drying operation. Furthermore, the use of perforated metal trays with 0.5 mm openings within the drying room promotes uniform hot air distribution over the product surface, thereby improving overall drying performance.

The fundamental working mechanism of the system is based on regulating the airflow path to ensure a consistent drying environment under elevated temperature conditions. Ambient air is supplied to the system by a fan and heated via solar energy while flowing through the centrally positioned copper tube. The resulting hot air is conveyed to the drying room, where it interacts with the product surface and promotes moisture evaporation. The moisture-laden air exits through the upper outlet of the chamber, maintaining continuous airflow, accelerating the drying process, and reducing overall energy demand.

The drying rate was regulated through a programmable logic controller (PLC). Within the experimental setup, the PLC was employed to adjust the fan speed, thereby governing the drying air velocity. Throughout all experimental runs, the airflow was kept constant at 1.5 m/s.

**Figure 2:**
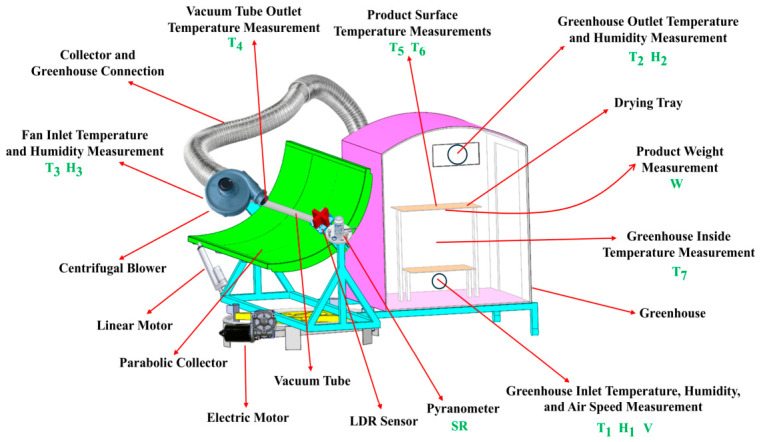
Schematic representation of greenhouse dryer.

Figure 2 presents the components, sensor measurement locations, and a schematic representation of the PTSC greenhouse dryer. The key elements of the system—namely the electric motor, fan, linear motor, vacuum tube, parabolic collector, greenhouse, supporting platform, and the connection line between the vacuum tube and the greenhouse—are clearly illustrated. As shown in the figure, the sensor measurements include the greenhouse air entry temperature and humidity (T_1_ and H_1_), air velocity (V), greenhouse air exit temperature and humidity (T_2_ and H_2_), weight measurement (W), fan air entry temperature and humidity (T_3_ and H_3_), which also represent the ambient conditions, the temperature measured at the vacuum tube exit (T_4_), surface temperature of the product measured by two sensors (T_5_ and T_6_), drying cabinet interior temperature (T_7_) and solar radiation (SR). In addition, the electrical power consumption is represented in watts. Table 1 presents the sensors employed in the experimental setup along with their technical specifications, including measurement ranges, sensitivities, and manufacturer information. The PLC system stored the experimental data in the data logger at a sampling rate of one record per second, enabling real-time monitoring of variations in the measured parameters.

**Figure 3:**
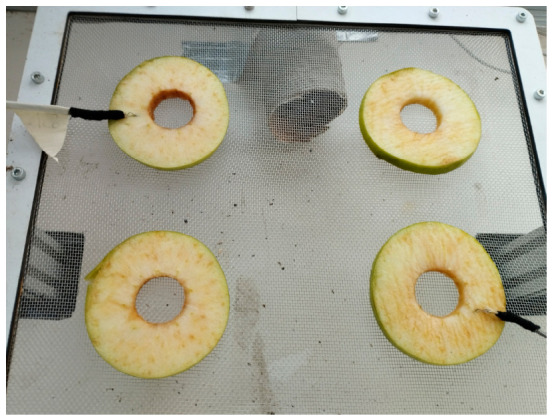
Drying samples.

The visual representation of the hollowed, oval-shaped apple slices and their uniform distribution on the perforated drying trays prior to the experiment is presented in Figure 3.

### 2.2. Energy and Exergy Equations

Energy efficiency (η_en_) is defined as the rate of the useful heat output (Q_u_) supplied by the collector to the solar radiation (I_b_) incident on the aperture area (A_a_), and it is calculated using Equation (1) for the greenhouse drying system [23].

(1) $$\eta_{\mathrm{en}}=\frac{Q_{u}}{A_{a}\cdot I_{b}}=\frac{\dot{m}\cdot c_{p}\cdot \Delta T}{A_{a}\cdot I_{b}}$$

Equation (2) is applied to determine the aperture area, where L_r_ denotes the collector’s mirror length and W_r_ represents the overall width of the rectangular opening.

(2) $$A_{a}=L_{r}\cdot W_{r}$$

Drying efficiency is a key indicator for assessing the operational effectiveness of a solar drying system and is expressed by Equation (3) [24]. To evaluate the drying efficiency, the total energy required for moisture evaporation from the product was taken into account. Accordingly, the overall energy entry included the electrical energy consumed by the system elements (E_T_), the thermal energy supplied by the solar collector (Q_s_), and the solar energy (Q_c_) transmitted through the greenhouse cover per unit time, which is calculated as the product of the greenhouse cover area and the incident solar radiation intensity per unit time. In this equation, the dryer outlet temperature is T_o_, the ambient reference temperature is T_a_, and the mass variation in the dried material is Δm.

(3) $$\eta_{d}=\frac{\left(m_{i}-m_{f}\right)\cdot \left(L_{w}+C_{\mathrm{pw}}\left(T_{o}-T_{a}\right)\right)}{E_{T}+Q_{s}+Q_{c}}$$

The exergy efficiency is determined with Equation (4), considering the solar temperature of 4350 K along with the operating entry and exit temperatures, where T_pi_ and T_po_ represent the PTSC’s entry and exit temperatures, respectively [17,25].

(4) $$\eta_{\mathrm{ex}}=\frac{\dot{m}C_{p}\left[\left(T_{\mathrm{po}}-T_{\mathrm{pi}}\right)-T_{a}\left(\frac{\ln T_{a}}{T_{\mathrm{pi}}}\right)\right]}{A_{P}I_{b}\left[1-\left(\frac{T_{a}}{T_{\mathrm{sun}}}\right)\right]}$$

In addition to the efficiency calculations, the physical interpretation of the XAI rules was evaluated in light of the governing heat and mass transfer mechanisms of the drying process. The energy-related rules were assessed considering the solar energy input, useful heat gain, and heat loss potential between the collector and the ambient environment. The drying-efficiency rules were interpreted in terms of moisture evaporation, vapor pressure difference between the product surface and drying air, and internal moisture diffusion resistance. The exergy-related rules were evaluated using the second-law framework, accounting for useful energy quality, dead-state conditions, and irreversibilities associated with heat and moisture transfer.

### 2.3. Economic and Practical Feasibility Analysis

In addition to the energy, exergy, and drying performance analyses, a preliminary economic and practical feasibility assessment was carried out to evaluate the applicability of the PTSC-integrated greenhouse dryer under seasonal operating conditions. The economic evaluation was based on the simple payback period method, which is commonly used as an initial screening indicator for renewable energy and energy-efficiency systems. This approach is suitable for small-scale solar thermal applications because it provides a direct estimate of the time required to recover the initial investment through annual operating cost savings [26,27].

To avoid arbitrary cost assumptions, the investment cost of the experimental system was estimated based on the actual prototype components and local market-based procurement prices. The main cost items included the parabolic trough solar collector, greenhouse drying chamber, vacuum tube/receiver unit, fan and air ducting system, PLC-control unit, temperature–humidity sensors, load cell, pyranometer, mechanical support frame, assembly, and auxiliary electrical components. The component-based cost breakdown used in the analysis is presented in Table 2. The total initial investment cost of the PTSC-integrated greenhouse dryer was determined as 1550 USD. This cost level is consistent with small-scale experimental solar drying systems reported in the literature, where the capital cost is mainly governed by the solar collector, control system, drying chamber, and supporting structure [28,29,30].

The economic benefit of the proposed system was evaluated relative to a conventional electrically heated dryer operating under the same drying load and drying period. Since the PTSC-integrated system supplies a considerable portion of the required thermal energy from solar radiation, the economic savings were defined as the avoided electrical heating costs after subtracting the auxiliary electricity demand of the fan, control unit, sensors, and data acquisition system. The annual operating cost saving was calculated as:

(5) $$S_{\mathrm{annual}}=S\times N$$

where S_annual_ is the annual operating cost saving, S is the operating cost saving for a single drying period, and N is the number of drying periods per year. The saving per drying period was calculated as:

(6) $$S=\left(E_{\mathrm{conv}}-E_{\mathrm{aux}}\right)\times C_{e}$$

where E_conv_ is the electrical energy that would be required by a conventional electrically heated dryer for the same drying process, E_aux_ is the measured auxiliary electrical energy consumption of the PTSC-integrated greenhouse dryer, and C_e_ is the local electricity price. In the present study, the unit electricity price was taken as 0.10 USD/kWh based on the local electricity tariff/invoice value during the experimental period. For seasonal operation, the system was assumed to operate for 120 drying periods per year, which corresponds to repeated use during the main fruit and vegetable drying season in regions with suitable solar radiation potential. Based on the measured/estimated reduction in conventional electrical heating demand, the average operating cost saving was determined as 5 USD per drying period.

The simple payback period was calculated as:

(7) $$\mathrm{PP}=\frac{C_{\mathrm{inv}}}{S_{\mathrm{annual}}}$$

where *PP* is the simple payback period and C_inv_ is the total initial investment cost of the PTC-integrated greenhouse dryer. In the base-case scenario, the total initial investment cost was taken as 1550 USD. The average operating cost saving was assumed to be 5 USD per drying period, based on the expected reduction in conventional electrical heating demand, and the system was assumed to operate for 120 drying periods per year during the seasonal drying period. Accordingly, the annual operating cost saving was calculated as 600 USD/year. Based on this annual saving, the simple payback period was obtained by dividing the initial investment cost by the annual operating cost saving, resulting in 2.58 years. Therefore, under the base-case operating conditions, the payback period of the proposed PTSC-integrated greenhouse dryer was estimated to be approximately 2.6 years.

To examine the effect of uncertainty in operating conditions, a simple sensitivity analysis was also performed. Since the economic performance of solar drying systems may vary depending on electricity price, annual drying frequency, crop type, solar radiation level, and actual utilization rate, three different saving scenarios were considered. When the operating cost saving was assumed as 3, 5, and 7 USD per drying period, the corresponding annual savings for 120 drying periods were 360, 600, and 840 USD/year, respectively. Under these conditions, the payback periods were calculated as 4.31, 2.58, and 1.85 years. These results indicate that the economic feasibility of the system is strongly affected by the seasonal utilization rate and the amount of conventional electrical energy replaced by solar thermal energy.

The present economic analysis should be considered as a preliminary feasibility assessment rather than a comprehensive life-cycle cost analysis. Maintenance cost, component replacement, inflation, discount rate, residual value, and product-market revenue were not included in the base-case calculation. Nevertheless, the obtained payback period indicates that the PTSC-integrated greenhouse dryer has practical economic potential for repeated seasonal use, particularly in regions with high solar radiation potential and frequent agricultural drying demand. Future studies should extend the economic evaluation by including net present value, internal rate of return, discounted payback period, maintenance cost, long-term performance degradation, and product-quality-based economic benefits. The main economic assumptions and input parameters used in the feasibility analysis are summarized in Table 2.

As shown in Table 2, the economic assessment was based on a component-based initial investment cost and a base-case seasonal operating scenario. The annual saving was estimated by multiplying the average operating cost saving per drying period by the assumed number of annual drying periods. Accordingly, the simple payback period was calculated using the ratio of the initial investment cost to the annual operating cost saving. These assumptions provide a transparent basis for evaluating the preliminary economic feasibility of the PTSC-integrated greenhouse dryer and allow the sensitivity of the payback period to be examined under different saving scenarios.

### 2.4. Chaotic Rule Based Strength Pareto Evolutionary Algorithm 2 (CRb-SPEA2)

In this study, the CRb-SPEA2 algorithm was employed to derive interpretable classification rules for the energy, drying, and exergy efficiency datasets [31]. The algorithm is based on the fundamental principles of SPEA2 and incorporates a chaotic mapping mechanism to improve the exploration of the solution space [32]. The motivation behind using CRb-SPEA2 in this study is twofold: to increase the variety of candidate solutions through Pareto fronting, and to leverage its ability to optimise multiple objectives. Traditional rule derivation techniques often optimise a single objective; however, due to their sequential, greedy search strategies, they can overlook alternative rule sets. However, CRb-SPEA2 can search for multiple non-dominant rule candidates simultaneously. Furthermore, using a Tent chaotic map instead of random number generation during the evolutionary process increases population diversity and reduces the probability of early convergence to local optima. This mechanism enables the discovery of more robust and interpretable decision rules. The algorithm begins the optimization process using an initially empty archive that is continuously updated along with a starting population. Two variables, termed “Strength” and “Raw fitness”, are utilised to show whether individuals exert dominance. In the context of multi-objective optimization, “dominance” refers to the situation where one solution performs better than another across multiple evaluation criteria simultaneously. The quantity known as Strength (St(j)), which represents the quantity of solutions outperformed by the jth individual in the tth iteration, is of particular significance (Equation (8)).

(8) $$\mathrm{St}\left(j\right)=\left|\left\{\left.i\right|i\in P_{t}+{\bar{P}}_{t}\wedge j≻i\right\}\right|$$

The raw fitness value (Equation (9)) is indicative of the number of times an individual is dominated. A high Rf (j) value indicates that the individual is heavily dominated, while a value of 0 indicates that the individual is not dominated at all.

(9) $$\mathrm{Rf}\left(j\right)=\sum_{i\in P_{t}+\bar{P_{t}},i≻j}\mathrm{St}(j)$$

Following the determination of the raw fitness and strength values, it is evident that these two results may not provide sufficient information to facilitate a comprehensive analysis of net optimization in the calculation of dominance value. In order to address this deficiency, the density (D(j)) value is calculated and the kNN method is used. Subsequent to the completion of these steps, the fitness value of the relevant individual is calculated using Equation (10).

(10) $$F\left(j\right)=\mathrm{Rf}\left(j\right)+D(j)$$

After the calculation of the fitness values of individuals, the subsequent generation is generated. Individuals whose fitness values are below 1 are incorporated into the archive until its capacity limit is reached. (Equation (11)).

(11) $${\bar{P}}_{t+1}=\left\{j\left|j\right.\in P_{t}+\bar{P_{t}}\wedge F(j)<1\right\}$$

In contrast to the SPEA2 algorithm, the CRb-SPEA2 algorithm (Equation (12)) incorporates the tent chaotic map as a component of its randomness generation mechanism [33].

(12) $$X_{n+1}=\left\{\begin{array}{ll} \partial X_{n}, & X_{n}<\frac{1}{2}\\ \partial \left(1-X_{n}\right), & \frac{1}{2}\leq X_{n} \end{array}\right\}$$

Furthermore, new sections have been incorporated into the SPEA2 algorithm to address the representation format and the evaluation of objectives. The utilisation of a predetermined threshold value facilitates the determination of the solution’s inclusion within the rule. The rules comprise two sections: The use of the conjunctions “if” and “then” is pivotal in this analysis. In Equation (13), the lower values of candidates 4 and 7 are denoted by $s_{4}^{l}$, $s_{7}^{l}$; respectively, while the upper values of the same candidates are denoted by $s_{4}^{u}$, $s_{7}^{u}$. The attribute F and the class C to which they belong are also denoted here. In accordance with the ‘if and then’ sections of the rule, the True Positive (TP), True Negative (TN), False Positive (FP), and False Negative (FN) values are calculated (Table 3).

(13) $$\mathrm{if}\;s_{4}^{l}\leq F_{4}\leq s_{4}^{u}\;\mathrm{and}\;s_{7}^{l}\leq F_{7}\leq s_{7}^{u}\;\mathrm{then}\;C$$

In this study, Accuracy (Acc), Precision (Pre) and Recall (Rec) values were taken as conflicting metrics (Equations (14)–(16)).

(14) $$\mathrm{Acc}=\frac{\mathrm{TP}+\mathrm{TN}}{\mathrm{TP}+\mathrm{TN}+\mathrm{FP}+\mathrm{FN}}$$

(15) $$\mathrm{Pre}=\frac{\mathrm{TP}}{\mathrm{TP}+\mathrm{FP}}$$

(16) $$\mathrm{Recall}=\frac{\mathrm{TP}}{\mathrm{TP}+\mathrm{FN}}$$

In the dataset, T_1_, T_2_, T_3_, T_4_, T_5_, H_1_, H_2_, H_3_, W, and SR shown in Figure 2 are used as input parameters. The programmable logic controller (PLC) recorded measurements at 1-s intervals, resulting in 32,400 data points over the 3-day experimental campaign conducted from 10:00 to 13:00. These data were structured as input–output pairs for the artificial intelligence (AI)-based classification process. Input variables included T1, T2, T3, T4, T5, H1, H2, H3, W, and SR, while output variables comprised the low, medium, and high classes of energy, drying, and exergy efficiencies. The dataset, consisting of a total of 32,400 data points, represents continuous monitoring rather than independent experimental replicates. A one-second sampling interval was selected in order to capture rapid variations in solar radiation, collector temperature and drying conditions, thereby preserving the transient thermodynamic behaviour necessary for rule extraction. As expected in dynamic thermal systems, consecutive observations exhibit temporal dependence. Nevertheless, each observation corresponds to a different operating state, which is defined by continuously varying solar radiation, temperatures, humidity levels and product weight. Using the complete dataset aimed to characterise the dynamic evolution of the drying process and derive interpretable operating rules across the entire operating range, rather than estimating statistical properties from independent observations.

## 3. Results

### 3.1. Experimental Results

**Figure 4:**
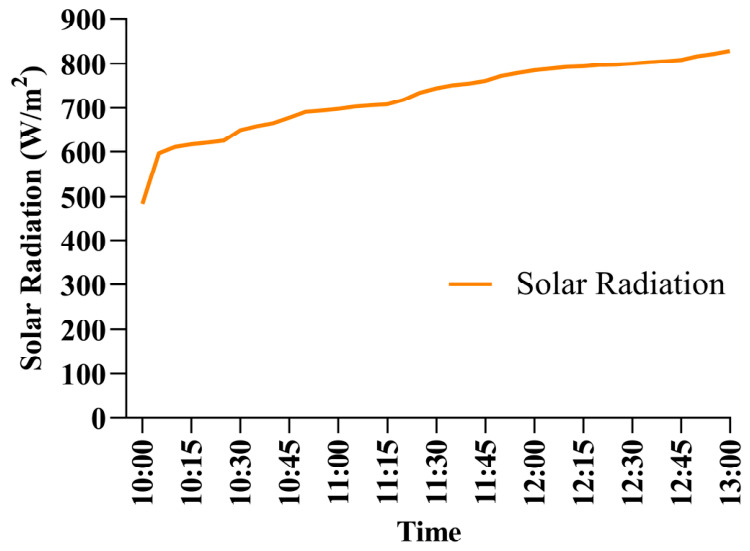
Change in solar radiation during the experiments.

Figure 4 illustrates the time-dependent variation in solar radiation over the course of the experiment. The variation in solar radiation during the measurement period exhibits a clear increasing trend from morning to early afternoon. In the morning hours, at 10:00, the solar radiation was measured as 484.3 W/m^2^, indicating moderate solar intensity at the beginning of the drying process. As time progressed toward noon, solar radiation increased steadily, reaching approximately 698 W/m^2^ around 11:00, which reflects a significant enhancement in solar availability. Near noon, the irradiance levels continued to rise and exceeded 780 W/m^2^ at 12:00, indicating strong solar conditions suitable for effective solar drying. The solar radiation reached its maximum value of 828.4 W/m^2^ at 13:00, representing the peak irradiance during the considered time interval. Overall, the mean solar radiation between 10:00 and 13:00 was calculated as 725.8 W/m^2^, demonstrating consistently high solar energy availability throughout the main drying interval.

**Figure 5:**
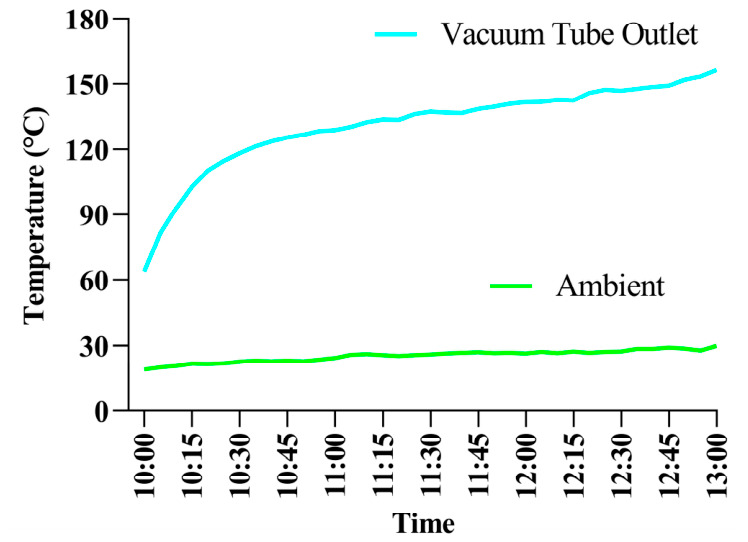
Change in ambient and vacuum tube outlet temperatures with respect to time.

Figure 5 shows the alteration of the vacuum tube exit temperature and the ambient temperature with time. The outlet temperature of the vacuum tube exhibited a pronounced increase from morning to early afternoon. At the beginning of the operation period, at 10:00, the outlet temperature was measured as 63.9 °C, indicating relatively low thermal energy collection during the early hours. As solar intensity increased, the outlet temperature rose rapidly, reaching approximately 133.5 °C around 11:15, which reflects a significant improvement in the thermal performance of the collector. At 12:15, the vacuum tube outlet temperature exceeded 142.3 °C, demonstrating strong thermal energy accumulation within the receiver. The maximum outlet temperature of 156.4 °C was recorded at 13:00, corresponding to peak solar conditions during the measurement interval. Over the entire period between 10:00 and 13:00, the average outlet temperature was calculated as 131.0 °C, indicating consistently high-temperature operation of the PTSC system.

When examining the ambient temperature, which is another result presented in Figure 5, the variation in environmental temperature during the experimental period shows a gradual increase from morning to early afternoon. At the beginning of the measurements, at 10:00, the ambient temperature was recorded as 19.1 °C, indicating relatively mild environmental conditions. As the day progressed, the ambient temperature increased steadily, reaching approximately 26 °C around 11:30, which reflects the warming of the surrounding environment due to increasing solar intensity. Toward midday, ambient temperature values continued to rise with minor fluctuations. At 12:30, the ambient temperature exceeded 27.4 °C, while the maximum value of 29.9 °C was observed at 13:00, corresponding to peak daytime conditions. Over the entire period between 10:00 and 13:00, the average ambient temperature was calculated as 25.3 °C, indicating moderate and stable environmental conditions during the experiments.

**Figure 6:**
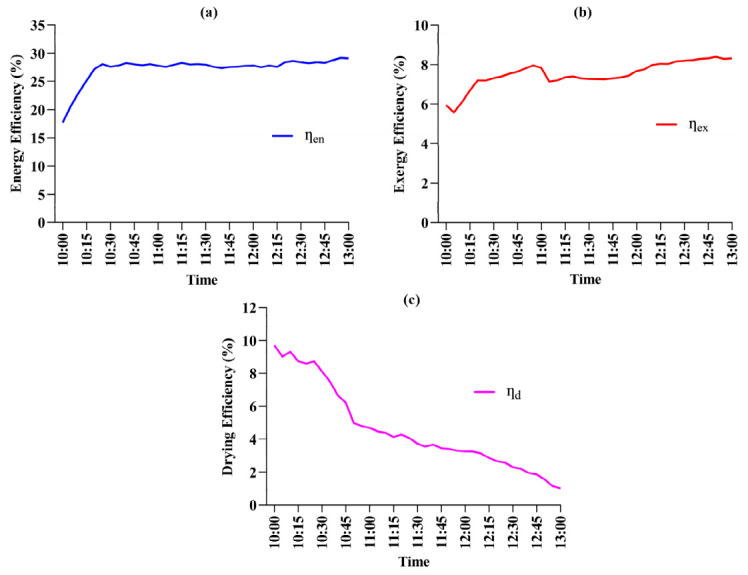
Time-dependent alterations in energy (**a**), exergy (**b**), and drying efficiencies (**c**).

Figure 6 presents the change in energy, exergy, and drying efficiencies during the experimental period. An examination of the energy efficiency results depicted in Figure 6a reveals that energy efficiency indicates a clear enhancement from the early morning toward noon throughout the experimental period. At 10:00, the energy efficiency was measured as 17.7%, reflecting relatively limited system performance at the beginning of operation. As solar input increased, energy efficiency rose noticeably and reached approximately 27.6% around 11:05, demonstrating a substantial improvement in the collector’s energy conversion capability. During the later hours, the energy efficiency exhibited minor fluctuations while maintaining relatively high values. At 12:05, the efficiency remained close to 27.6%, indicating sustained system performance under increased thermal input. The highest energy efficiency of 29.2% was recorded at 12:55, followed by a value of 29.1% at 13:00, representing peak operational efficiency during the measurement interval. Over the period between 10:00 and 13:00, the average energy efficiency was calculated as 27.4%, confirming that the system operated predominantly within a high-efficiency range.

An analysis of the exergy efficiency outputs presented in Figure 6b shows that exergy efficiency improves from the early operating hours toward noon over the course of the experiment. At 10:00, the exergy efficiency was determined as 5.95%, indicating limited thermodynamic performance under low solar input conditions. With increasing solar availability, the exergy efficiency increased notably and reached approximately 7.39% around 11:20, demonstrating enhanced utilization of the available energy potential. In the subsequent period, the exergy efficiency remained within a relatively narrow range with moderate variations. At 12:20, exergy efficiency increased to 8.03%, followed by further improvement as the system approached peak operating conditions. The maximum exergy efficiency of 8.39% was observed at 12:50, while a comparable value of 8.33% was recorded at 13:00. Considering the entire interval between 10:00 and 13:00, the average exergy efficiency was calculated as 7.5%, indicating sustained thermodynamic performance during the main operating period.

An investigation of the drying efficiency findings given in Figure 6c indicates that drying efficiency exhibited a decreasing tendency throughout the experimental period. At the beginning of the drying process, at 10:00, the drying efficiency was measured as 9.72%, representing the highest value observed during the entire operation. As the process continued, the drying efficiency declined progressively, reaching approximately 4.68% around 11:00, which indicates a substantial reduction compared to the initial stage. During the later hours, the drying efficiency continued to decrease as the product’s moisture content diminished. At 12:00, the efficiency dropped to 3.26%, while the minimum value of approximately 1.00% was recorded at 13:00, corresponding to the final stage of the drying process. Considering the full duration between 10:00 and 13:00, the average drying efficiency was calculated as 4.57%.

The relatively low ηd values observed in this study are primarily attributed to the comprehensive energy input considered in Equation (3). In this context, ηd reflects a general system-based drying efficiency rather than solely local evaporation efficiency, as the denominator encompasses the total energy supplied to the drying process. This situation includes the electrical energy consumed by system components, the thermal energy provided by the collector, and the solar energy transmitted through the greenhouse cover. Consequently, ηd values ranging from 1.0% to 9.7% should be interpreted within this broader framework of energy balance. Comparable low drying efficiency values have been reported in previous studies. For example, Ferreira et al. reported drying efficiencies of 5.2% to 7.2% for an active integrated solar dryer, while their instantaneous thermal efficiencies ranged from 9.7% to 29.5% [34]. Gilago et al. found average drying efficiencies of 7.5% and 10.25% for passive indirect solar dryers without and with thermal energy storage, respectively [35]. Susana et al. also noted that drying efficiency decreases throughout the drying process, as the energy absorbed by the product diminishes with decreasing moisture content [36]. Thus, the low and decreasing ηd values observed in this study align with the general system-based efficiency definition and the characteristic drying behavior at reduced moisture removal rates.

### 3.2. Explainable Artificial Intelligence Results

The present study examined the performance metrics and rules for the drying, energy, and exergy efficiency datasets. The data were then categorised into three ranges: High (H), Medium (M), and Low (L). Before the rule inference process, each dataset was randomly divided into 70% training and 30% testing subsets. The training subset was used for evolutionary rule generation and optimization, whereas the testing subset was reserved exclusively for performance evaluation. Since CRb-SPEA2 is a stochastic evolutionary optimization algorithm, the complete optimization procedure was repeated 20 independent times using different random seeds while keeping all algorithm parameters unchanged. For each independent run, the best-performing rule obtained on the test set was selected, and the corresponding Accuracy, Precision and Recall values were recorded. The robustness of the proposed approach was evaluated by reporting the mean, median, standard deviation of these performance metrics over the 20 independent runs. The Python 3.14 programming language was utilised to derive rules and calculate performance values, and WEKA (Version 3.8.6) was employed to compare with traditional machine learning algorithms. The ensuing algorithms were utilised for the purpose of comparison: Multilayer Perceptron (MLP), Support Vector Machine (SVM), IBk (kNN Nearest Neighbor), and Naive Bayes (NB). In comparisons with classical machine learning algorithms, standardized parameter settings were used to provide a consistent benchmark framework. The aim is not extensive hyperparameter optimization of black box models, but rather to evaluate their predictive behavior and interpretability performance under comparable application conditions.

#### 3.2.1. Drying Efficiency Prediction Results

Table 4 presents the rules developed for drying efficiency. The generated rules indicate that, for the H category, drying efficiency depends on weight, greenhouse inlet relative humidity, and solar radiation, whereas for the M category, it is associated with weight, greenhouse inlet relative humidity, greenhouse outlet temperature, and solar radiation. For the L category, drying efficiency is governed by weight, greenhouse inlet relative humidity, greenhouse outlet temperature, and product surface temperature.

According to the rules in Table 4, the variables W (weight or weight change, measured by load cell) and H1 (relative humidity) are most important in judging drying efficiency (η_d), along with SR and the levels of T2/T5. In the ‘high efficiency’ rules, W, either alone or with SR/T2, matches drying efficiency in certain ranges, as shown directly by the amount of moisture lost (weight loss). On the other hand, H1 is more common in the ‘low efficiency’ rules, and T2/T5 are distributed across lower ranges, indicating that η_d decreases when air can hold less moisture and the driving force for evaporation weakens.

These results are in line with studies showing that thermal behavior of greenhouse-type solar drying systems is dependent on how sunlight, temperature, humidity, and air movement interact. These factors influence drying speed and energy use [37]. Also, experiments show that drying works better at higher collector temperatures in PTC-supported greenhouse setups, which matches what is shown on the SR–T2/T5 axis, as seen in Table 4 [38,39].

**Figure 7:**
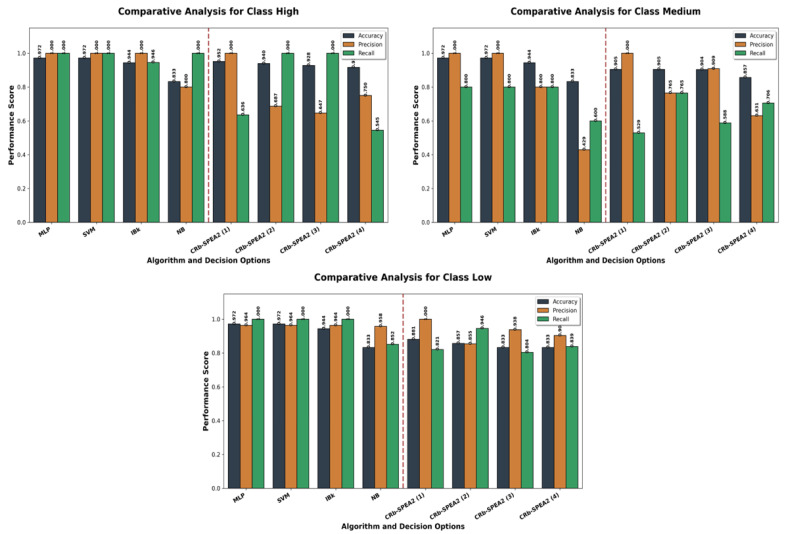
Comparative performance of CRb-SPEA2 and baseline ML models for drying efficiency classes.

Figure 7 shows the comparison of operational efficiency of the proposed CRb-SPEA2 algorithm with the MLP, SVM, IBk, and NB algorithms for the drying efficiency dataset. In consideration of the drying efficiency data set, it is evident that the MLP and SVM methods yielded optimal outcomes with Acc, Pre, and Rec values of 0.972, 1.000, and 1.000 for class H. However, these methods were unable to surpass the CRb-SPEA2 method, which produced results of 0.940, 0.687, and 1.000. For class M, the MLP and SVM methods achieved results of 0.972, 1.000, and 0.800, while the CRb-SPEA2 algorithm also demonstrated efficacy with results of 0.905, 1.000, and 0.529. In the context of class L, and once more within the framework of classical algorithms based on machine learning, multi-layer perceptron (MLP) and support vector machine (SVM) algorithms achieved success, with values of 0.972, 0.964 and 1.000. Concurrently, the CRb-SPEA2 algorithm yielded results that were competitive with those of classical machine learning methods, with values of 0.881, 1.000 and 0.821.

#### 3.2.2. Energy Efficiency Dataset

Table 5 includes the rules generated for energy efficiency. The derived rules show that, for the H class, energy efficiency relies on ambient temperature and solar radiation, whereas for both M and L classes, it is related to solar radiation, vacuum tube outlet temperature, ambient temperature, and ambient relative humidity.

The energy efficiency rules sets presented in Table 5 (η_en) rule sets show that the high efficiency (H) class predominantly occurs in ranges where ambient temperature (T3) and solar radiation (SR) increase together; in the medium and low efficiency (M–L) classes, in addition to SR, vacuum tube outlet temperature (T4) and ambient relative humidity (H3) become decisive. The differentiation of the rules derived for the H class based on T3–SR conditions (e.g., transition to the H class with high SR in a specific T3 band) indicates that energy efficiency is fundamentally shaped by radiation-induced heat input and environmental loss terms (temperature difference related to T3). In contrast, the inclusion of T4 and H3 in the rules for the M–L classes is consistent with the differentiation of the system’s heat production/transport capacity and heat losses under humid ambient conditions. This trend is consistent with studies reporting that efficiency in PTSC is strongly associated with solar radiation and environmental temperature, and that losses can limit efficiency as temperature levels (outlet/operating temperature) increase [5,40]. Furthermore, the literature demonstrates that the energy/exergy performance of greenhouse-type dryers is sensitive to irradiation and ambient temperature; therefore, the T3–SR weighted separation in Table 5 is an expected result [41].

**Figure 8:**
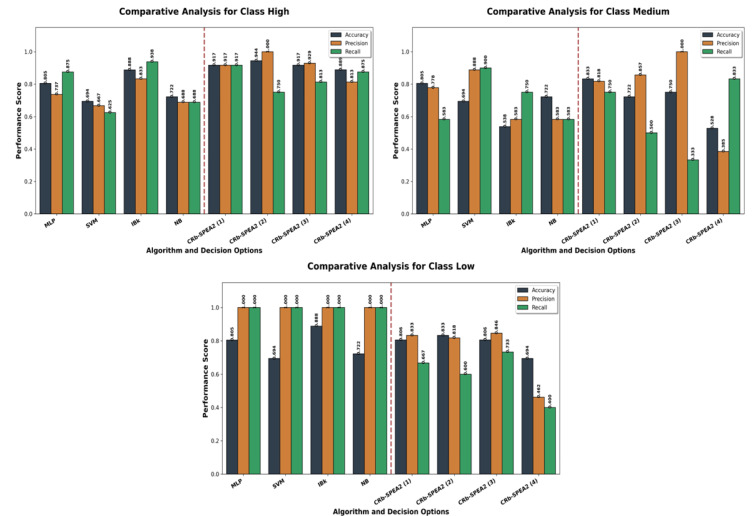
Comparative performance of CRb-SPEA2 and baseline ML models for energy efficiency classes.

Figure 8 provides a performance comparison of the proposed CRb-SPEA2 algorithm and the MLP, SVM, IBk, and NB algorithms for the energy efficiency dataset. In the context of the energy efficiency dataset, it was observed that the conventional machine learning methodologies belonging to the H class did not demonstrate superior performance regarding Accuracy, Precision, and Recall when evaluated against the CRb-SPEA2 algorithm. The CRb-SPEA2 algorithm showed robust operational behavior, with an Acc value of 0.964, a Pre value of 1.000, and a Rec value of 0.952, indicating its resilience to domination by other methods. In the context of classical machine learning methodologies, IBk has been demonstrated to consistently generate optimal outcomes. For the M class, although the IBk algorithm yielded the best accuracy with an Acc value of 0.888, no classical machine learning algorithm dominated CRb-SPEA2 across all metrics. Upon rigorous examination of the L class, it was determined that the IBk algorithm exhibited a predominant presence, surpassing all other algorithms in terms of its prevalence.

#### 3.2.3. Exergy Efficiency Dataset

Table 6 indicates the rules created for exergy efficiency. The rules specify that, for the H group, exergy efficiency is connected to the vacuum tube outlet temperature, environmental temperature, and solar radiation. On the other hand, vacuum tube outlet temperature, ambient relative humidity, solar radiation and surrounding temperature are the parameters that are effective in deriving the rules for both M and L groups.

The exergy efficiency (η_ex) rules in Table 6 indicate that the classification is primarily formed around the vacuum tube outlet temperature (T4); in class H, T4 alone can be a distinguishing factor within a high range, and in some rules, the ambient temperature (T3) also plays a threshold role alongside SR. In contrast, in classes M and L, the more frequent inclusion of ambient relative humidity (H3) and SR in the rules, along with T4, indicates that exergy efficiency is affected not only by temperature level but also by heat losses and the quality of usable energy, determined by environmental conditions. This trend is consistent with the literature: it has been reported that exergy efficiency in solar dryers varies significantly with air/system temperature levels and solar input, and that environmental conditions (particularly temperature and humidity) affect exergy destruction and sustainability indicators [42]. Similarly, it has been demonstrated that operating/output temperature and irradiance are fundamental parameters determining exergy performance in PTSC and can impose limitations due to heat losses; the divergence observed along the T4–SR axis in Table 6 supports this physical framework [43].

The CRb-SPEA2 rules should be interpreted not only as data-driven threshold intervals, but also as physically meaningful operating regions. For ηd, the appearance of W, H1, SR, T2, and T5 is consistent with the moisture removal mechanism, since W reflects the amount of evaporated moisture, H1 controls the vapor pressure difference between the product surface and drying air, and SR–T2–T5 represent the thermal conditions governing evaporation. For ηen, the dominance of SR, T3, and T4 agrees with the collector energy balance, where SR is the main energy input, T4 represents useful thermal gain, and T3 affects heat losses to the surroundings. Similarly, for ηex, the frequent occurrence of T4, SR, T3, and H3 is consistent with second-law behavior, as exergy efficiency depends on both the temperature of the useful heat and the ambient/dead-state conditions. Therefore, the rule intervals in Table 4, Table 5 and Table 6 indicate physically interpretable operating windows governed by heat transfer, mass transfer, and exergy destruction mechanisms rather than purely statistical thresholds.

It has been established that CRb-SPEA2 is a stochastic evolutionary optimisation algorithm. Consequently, it is reasonable to hypothesise that the performance of the algorithm may vary between independent executions. Therefore, the proposed method was executed 20 independent times using different random seeds while maintaining identical algorithmic parameters. For each execution, the rule that demonstrated the highest level of performance on the test set was selected. Table 7 summarizes the descriptive statistics of the obtained Accuracy, Precision, and Recall values, demonstrating that the proposed CRb-SPEA2 model maintains consistent performance stability across repeated independent executions. The observation of relatively small standard deviations in the majority of datasets indicates consistent optimisation behaviour. The presence of larger variations for some minority classes can be attributed to class imbalance and the stochastic nature of the evolutionary search process, rather than systematic instability of the proposed method.

As illustrated in Table 7, the descriptive statistics were obtained from 20 independent executions of the proposed CRb-SPEA2 algorithm. As CRb-SPEA2 employs a stochastic evolutionary search mechanism, it was necessary to perform repeated executions in order to evaluate the stability and consistency of the generated classification rules. The relatively minor variations observed for the majority of datasets indicate that the proposed framework produces consistent predictive performance across independent iterations while maintaining competitive classification accuracy.

**Figure 9:**
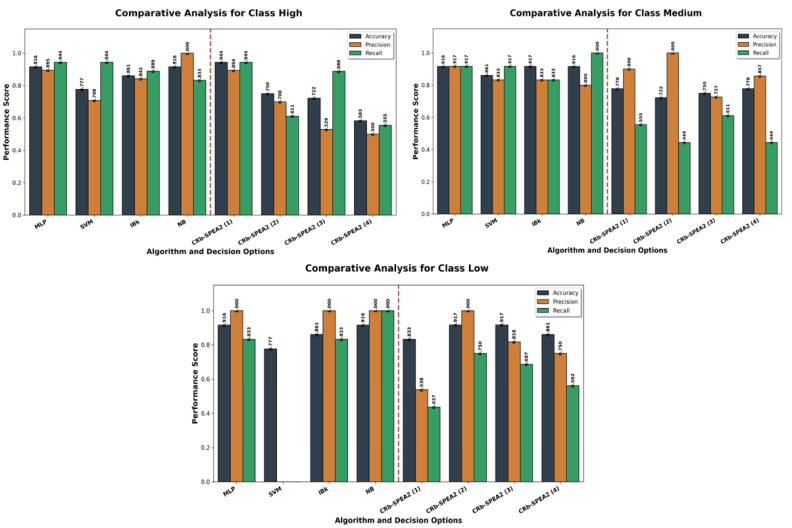
Comparative performance of CRb-SPEA2 and baseline ML models for exergy efficiency classes.

Figure 9 illustrates the comparative performance of the proposed CRb-SPEA2 method relative to the MLP, SVM, IBk, and NB algorithms using the exergy efficiency dataset. In the exergy efficiency dataset, the H class was found to be most effectively managed by the MLP and NB algorithms, with the NB algorithm achieving the highest Acc value of 0.916, the MLP achieving the highest Pre value of 1.000, and the CRb-SPEA2 algorithm achieving the highest Rec value of 0.971. Following a comprehensive analysis of all pertinent metrics, it was determined that CRb-SPEA2 did not demonstrate any signs of being dominated. A subsequent examination of the values in the M class revealed that the MLP and NB algorithms achieved an Acc value of 0.988, the CRb-SPEA2 algorithm attained a Pre value of 1.000, and the NB algorithm achieved a Rec value of 1.000. For the L class, the CRb-SPEA2 algorithm attained an Acc value of 0.988, the CRb-SPEA2, MLP, IBk, and NB algorithms attained a Pre value of 1.000, and the NB algorithm with the Rec value of 1.000 yielded successful results. Upon consideration of the aggregate results, it was determined that the CRb-SPEA2 algorithm demonstrated a high degree of success.

### 3.3. Economic Feasibility Result

Based on the preliminary economic assumptions defined in the methodology, the economic feasibility of the PTC-integrated greenhouse dryer was also evaluated. The total initial investment cost of the system was set at 1550 USD, and a unit electricity price of 0.10 USD/kWh was used to define the assumed operating cost savings relative to a conventional electrically heated dryer. For seasonal operation, the system was assumed to operate for 120 drying periods per year, with an average operating cost saving of 5 USD per drying period compared with a conventional electrically heated dryer. Accordingly, the annual cost saving was calculated as S_annual_ = 5 × 120 = 600 USD/year. Based on this value, the simple payback period was determined as PP = C_inv_/S_annual_ = 1550/600 = 2.58 years. Therefore, the payback period of the proposed PTC-integrated greenhouse dryer was estimated to be approximately 2.6 years. This result indicates that, despite the relatively high initial investment cost, the proposed system can reduce operating costs during repeated seasonal use by replacing a considerable portion of conventional electrical heating energy with solar thermal energy.

### 3.4. Limitations and Future Work

The findings of this study are based on experiments conducted in Tokat (Turkey) between 15 and 17 August 2023, with results reported as the average of three days. Therefore, they do not fully reflect how performance changes across different seasons and varying meteorological conditions (particularly cloud cover and humidity profiles). The drying experiments focused on a single product using a specific sample geometry: approximately 150 g of apple slices, each 15 mm thick. The air velocity was kept constant at 1.5 m/s across all conditions, and the collector outlet temperature was maintained between 60 and 80 °C. These parameters may restrict the applicability of the rule-based thresholds to different product loads, slice thicknesses, and air velocities. Despite the high recall values obtained in some of the classes, these results should be evaluated considering the relatively experimental conditions and the rule-based nature of the CRb-SPEA2 method. In addition to the high-parameter nature of black-box models, the proposed method produces physically interpretable threshold-based rules that reduce the tendency towards memorization. However, the absence of perfect precision values simultaneously in rules with high recall values indicates that the model does not memorize all samples. Future work should extend the dataset to different seasons and wider operating ranges (particularly air velocity and outlet temperature set points), conduct repeated experiments with different product types and slice thicknesses, and test the derived XAI rules with external validation on independent days/fields and similarly scaled systems, thereby enhancing the transferability of the rules while obtaining more robust operating limits for industrial applications.

## 4. Conclusions

This study involved apple drying experiments carried out in a greenhouse dryer integrated with a PTSC, and the results for key performance metrics, including energy, drying, and exergy efficiencies, are reported using both experimental and explainable artificial intelligence approaches. The most significant findings obtained are summarized below.

(1) The intensity of solar radiation peaks at 828.4 W/m^2^ at 13:00. In addition, the highest vacuum tube exit temperature of 156.4 °C occurred at 13:00, coinciding with the period of maximum solar radiation.
(2) Considering the complete drying duration from 10:00 to 13:00, the mean energy efficiency was determined to be 27.4%, the mean exergy efficiency was found to be 7.5%, and the mean drying efficiency was detected to be 4.57%.
(3) The CRb-SPEA2 explainable artificial intelligence algorithm achieved Recall scores of 0.952, 1.0, and 0.971 for energy, drying, and exergy efficiency rules, respectively, in the high-efficiency group. Although some baseline models achieved higher values for individual metrics in certain classes, CRb-SPEA2 provided competitive overall performance while additionally offering interpretable rule extraction.
(4) The interpretable rule sets derived using artificial intelligence based on the data obtained from the experiments clearly demonstrated that the variation in drying/energy/exergy efficiencies was fundamentally shaped by SR and system temperature levels (T2–T5), H1/H3 relative humidity conditions, and W (weight change), thus converting the experimental findings into physically consistent and applicable operating limits via decision rules.
(5) A preliminary economic feasibility assessment indicated that the PTC-integrated greenhouse dryer can be economically viable for seasonal use. Based on an initial investment cost of 1550 USD, an assumed operating cost saving of 5 USD per drying period, and 120 drying periods per year, the annual cost saving was calculated to be 600 USD. Accordingly, the simple payback period was estimated as approximately 2.6 years, indicating that the proposed solar-assisted drying system can reduce operating costs by partially replacing conventional electrical heating demand with solar thermal energy.

This study presents an innovative approach that generates multiple rules using explainable artificial intelligence to maximize the performance parameters of PTSC drying units, thereby distinguishing it from prior studies. The findings demonstrate that these rules can support the identification of favorable operating ranges for improving the energy, exergy, and drying performance of such systems. In this regard, the present study serves as a guide for researchers and manufacturing companies working on solar drying to optimize operational parameters.

## Figures and Tables

**Table 1:** Details of the sensors used in experimental setup.

Sensor Type	Measurement Interval	Manufacturer	Accuracy
Air Velocity	0 to 20 m/s	HK Instruments	±0.2 m/s
PT100	−50 °C to 205 °C	Baumer	±0.3 °C
Pyranometer	0 to 2000 W/m^2^	Kipp & Zonen	±0.2 W/m^2^
Load cell	10 kg	Zemic	±0.5 mV/V
Temperature and Relative Humidity	−40 °C to 125 °C and 0 to 100%	DFROBOT	±0.3 °C and ±3%

**Table 2:** Economic assumptions used in the feasibility analysis.

Parameter	Value	Basis
Initial investment cost, (Cinv)	1550 USD	Component-based prototype cost and local fabrication prices
Operating cost saving, (S)	5 USD/period	Estimated avoided electrical heating cost
Annual drying periods, (N)	120 periods/year	Seasonal agricultural drying assumption
Annual saving, (Sannual)	600 USD/year	(5 × 120)
Simple payback period, (PP)	2.58 years	(1550/600)
Sensitivity range	3–7 USD/period	Low, base, and high saving scenarios

**Table 3:** Calculation of TP, TN, FP and FN.

“if” Part	“then” Part	Operation
True	True	increase TP by 1
False	False	increase TN by 1
True	False	increase FP by 1
False	True	increase FN by 1

**Table 4:** Derived rules for drying efficiency dataset.

Class	Rule	Accuracy	Precision	Recall
H	if 122.018 < W < 145.865 then H	0.952	1	0.636
	if 41.377 < H1 < 52.386 then H	0.940	0.687	1
	if 102.549 < W < 148.300 then H	0.928	0.647	1
	if 417.899 < SR < 495.566 then H	0.917	0.750	0.545
M	if 33.329 < H1 < 46.733 and 54.577 < W < 83.857 then M	0.905	1	0.529
	if 65.131 < T2 < 81.109 and 28.777 < H1 < 49.455 and 725.973 < SR < 864.503 then M	0.905	0.765	0.765
	if 28.858 < H1 < 52.354 and 45.221 < W < 89.378 and 417.511 < SR < 851.437 then M	0.904	0.909	0.588
	if 26.293 < H1 < 52.265 and 48.231 < W < 97.263 and 420.399 < SR < 849.516 then M	0.857	0.631	0.706
L	if 30.857 < T2 < 71.388 and 15.226 < H1 < 40.700 then L	0.881	1	0.821
	if 15.254 < H1 < 40.663 then L	0.857	0.855	0.946
	if 28.136 < T2 < 65.117 and 36.355 < W < 125.222 then L	0.833	0.938	0.804
	if 33.812 < T5 < 76.456 then L	0.833	0.904	0.839

**Table 5:** Derived rules for energy efficiency dataset.

Class	Rule	Accuracy	Precision	Recall
H	if 21.876 < T3 < 28.899 and 784.397 < SR < 843.399 then H	0.964	0.950	0.905
	if 21.729< T3 < 29.716 and 798.780 < SR < 851.634 then H	0.940	1	0.762
	if 21.278 < T3 < 29.289 and 791.699 < SR < 849.919 then H	0.940	0.900	0.857
	if 21.864 < T3 < 28.962 and 776.256 < SR < 838.719 then H	0.940	0.833	0.952
M	if 112.647 < T4 < 156.442 and 576.480 < SR < 864.503 then M	0.821	0.833	0.714
	if 26.476 < T3 < 32.980 and 118.981 < T4 < 161.013 and 612.621 < SR < 843.718 then M	0.786	0.947	0.514
	if 26.445 < T3 < 32.980 and 123.465 < T4 < 164.176 and 607.509 < SR < 847.230 then M	0.774	1	0.457
	if 62.285 < T4 < 171.778 and 21.568< H3 < 36.942 then M	0.571	0.492	0.914
L	if 87.709 < T4 < 118.502 and 580.664 < SR < 790.581 then L	0.869	0.870	0.714
	if 28.155 < H3 < 34.981 and 556.475 < SR < 796.092 then L	0.821	0.842	0.571
	if 22.608 < T3 < 29.162 and 76.588 < T4 < 120.032 then L	0.821	0.883	0.536
	if 80.417 < T4 < 142.813 and 23.159 < H3 < 41.887 then L	0.726	0.568	0.750

**Table 6:** Derived rules for exergy efficiency dataset.

Class	Rule	Accuracy	Precision	Recall
H	if 125.354 < T4 < 179.760 then H	0.905	0.846	0.943
	if 21.297 < T3 < 28.461 and 76.643 < T4 < 175.556 and 729.939 < SR < 838.561 then H	0.774	0.786	0.629
	if 100.344 < T4 < 177.344 then H	0.702	0.586	0.971
	if 100.567 < T4 < 173.584 then H	0.583	0.500	0.686
M	if 64.249 < T4 < 127.239 and 24.284 < H3 < 41.306 then M	0.798	0.960	0.600
	if 22.888 < T3 < 30.717 and 22.272 < H3 < 38.636 and 409.859 < SR < 792.526 then M	0.738	1	0.450
	if 93.411 < T4 < 140.153 and 495.656 < SR < 793.542 then M	0.738	0.750	0.675
	if 22.802 < T3 < 29.665 and 86.399 < T4 < 152.844 then M	0.714	0.900	0.450
L	if 82.837 < T4 < 93.831 and 22.214 < H3 < 40.015 then L	0.893	0.500	0.444
	if 26.802 < T3 < 32.529 and 70.355 < T4 < 134.890 and 450.766 < SR < 641.584 then L	0.988	1	0.889
	if 21.798 < H3 < 24.320 and 381.049 < SR < 685.129 then L	0.964	0.875	0.777
	if 30.497 < T3 < 31.832 and 379.592 < SR < 695.748 then L	0.952	0.857	0.666

**Table 7:** Descriptive statistics of experimental results.

	ND-L			ND-M			ND-H			NEN-L			NEN-M			NEN-H			NEX-L			NEX-M			NEX-H		
	Acc	Pre	Rec	Acc	Pre	Rec	Acc	Pre	Rec	Acc	Pre	Rec	Acc	Pre	Rec	Acc	Pre	Rec	Acc	Pre	Rec	Acc	Pre	Rec	Acc	Pre	Rec
Mean	0.786	0.850	0.786	0.880	0.867	0.664	0.915	0.819	0.589	0.795	0.766	0.608	0.760	0.880	0.540	0.906	0.900	0.737	0.931	0.762	0.629	0.749	0.883	0.559	0.750	0.802	0.659
Median	0.822	0.854	0.813	0.893	0.893	0.692	0.917	0.775	0.523	0.805	0.789	0.595	0.757	0.892	0.500	0.905	0.937	0.707	0.943	0.789	0.750	0.754	0.899	0.540	0.738	0.789	0.625
Std. Dev.	0.081	0.089	0.145	0.030	0.141	0.197	0.022	0.184	0.259	0.046	0.148	0.114	0.052	0.125	0.141	0.037	0.118	0.191	0.037	0.214	0.250	0.028	0.093	0.107	0.071	0.158	0.207

## Data Availability

The data supporting the findings of this study are available from the corresponding author upon reasonable request.
